# Extraordinary Creatinine Level: A Case Report

**DOI:** 10.7759/cureus.9076

**Published:** 2020-07-08

**Authors:** Abuzar A Asif, Habiba Hussain, Tulika Chatterjee

**Affiliations:** 1 Internal Medicine, University of Illinois College of Medicine, Peoria, USA

**Keywords:** creatinine, chronic kidney disease, hemodialysis, uremia, end stage renal disease, hypertension

## Abstract

Creatinine, an amino acid derived from creatine, has been traditionally used to assess kidney function. However, its levels are significantly affected by nutritional status, muscle mass, age, and sex of an individual. The effect of creatinine levels on human physiology is not completely understood, and no correlation has been established between high creatinine levels and physiological equilibrium. We describe a case of a 27-year-old Hispanic male who presented with extremely elevated serum creatinine level (>37 mg/dL) with minimal symptoms of uremia and relatively fair functional status, eventually requiring hemodialysis. To our knowledge, based on a thorough review of the literature using PubMed, Medline, and Google Scholar, only four other cases have been reported with a creatinine level higher than that of our patient. A brief discussion on the utility of serum creatinine levels to assess mortality is provided using examples from similar case reports.

## Introduction

Approximately 14.8% of the United States population is affected by chronic kidney disease (CKD) [1]. Early diagnosis, determination of etiology, and appropriate intervention are vital to prevent progression to end-stage renal disease (ESRD) and development of cardiovascular comorbidities [1,2]. Challenges faced in timely diagnosis are the asymptomatic nature in early stages and the subtle clinical signs of advanced disease. The American College of Physicians and the United States Preventive Services Task Forces do not recommend screening of asymptomatic general population for CKD, but people with risk factors such as hypertension (HTN), diabetes, and family history of kidney disease should be screened annually with serum creatinine levels, urine microalbumin/creatinine ratio, and urine analysis [1].

## Case presentation

A 27-year-old Hispanic male with a past medical history of obesity (BMI: 28.59 kg/m²), long-standing uncontrolled HTN, stage 3 CKD, and obstructive sleep apnea presented to the hospital with complaints of fatigue, generalized weakness, and bleeding from the upper lip. Approximately a week ago, he got a cut on his lip while drinking from an open can and since then was suffering from slow oozing of blood from the wound site. He also reported 25-30 pounds unintentional weight loss over the past two months. He denied dysuria, hematuria, urgency, cloudy urine, abdominal pain, diaphoresis, fever, chills, syncopal attacks, palpitations, headache, blurry vision, dyspnea, or lower extremity swelling.

The patient was diagnosed with HTN at the age of 13. Three years ago, he was evaluated for exertional chest pain and uncontrolled HTN, which led to the diagnosis of CKD stage III/IV with a baseline creatinine of 2.9 mg/dL. Cortisol level was normal, aldosterone level was less than 1 ng/dL, and renin activity level was elevated at 24.40 ng/mL/hour, but the patient was on lisinopril and repeat testing after discontinuing lisinopril was normal at 1.4 ng/mL/hour. Vasculitis work-up including antinuclear antibody (ANA), anti-neutrophilic cytoplasmic antibody (ANCA) screen, anti-double stranded DNA antibody, and C3 and C4 complement levels were all within the normal range. Renal artery duplex had shown no signs of renal artery stenosis, and CT of the chest had shown a 3.7 cm (borderline) ascending thoracic aorta but no signs of coarctation. Transthoracic echocardiography findings were within the normal range. Exercise cardiac stress test showed no evidence of myocardial ischemia. No secondary cause of HTN could be elicited, and the patient was diagnosed with primary essential HTN. He was placed on three anti-hypertensive drugs: amlodipine 10 mg daily, carvedilol 25 mg two times a day, and hydralazine three times a day. He had stopped taking his blood pressure (BP) medication about one year ago due to insurance issues.

He reported consumption of two beers a week but denied smoking and recreational drug abuse. The patient was adopted at the age of 6 when he immigrated from Mexico to the United States; hence, substantial medical history of his biological family could not be obtained.

On arrival, the patient was afebrile with temperature of 98.7°F, respiratory rate of 14 breaths per minute, heart rate of 98 beats per minute, elevated BP of 175/99 mm Hg, 100% oxygen saturation at room air, and BMI of 27.12 kg/m². Systolic BP in his previous outpatient visits ranged from 140 to 180 mm Hg, indicative of poorly controlled BP. On physical examination, the patient appeared well built but he was pale and lethargic. He had a scab on his upper lip and on removal of the scab, slow oozing of blood was noted. Rest of his examination including cardiovascular, respiratory, abdominal, and neurological showed no abnormalities. Blood chemistry revealed serum sodium of 137 mmol/L, potassium of 5.0 mmol/L, chloride of 98 mmol/L, bicarbonate of 9 mmol/L, anion gap of 30 mmol/L, serum phosphorus of 11.7 mg/dL, corrected calcium of 7 mg/dL, blood urea nitrogen (BUN) of 228 mg/dL, and serum creatinine of >37 mg/dL (Architect Analyzer, Abbott Laboratories, Abbott Park, IL, USA, alkaline picrate kinetic [AP-K] method) [3]. The glomerular filtration rate (GFR) was unmeasurable and therefore not reported. The calculated GFR using the Modification of Diet in Renal Disease (MDRD) equation was less than 1.5 mL/minute/1.73 m². His baseline creatinine was around 2.9 mg/dL. Complete blood count showed hemoglobin of 4.7 g/dL, hematocrit of 15.8%, mean corpuscular volume of 87.3 fL, and platelet count of 127 x 10^3^/mcL. Peripheral smear showed poikilocytosis, ovalocytes, and elliptocytes. Parathyroid hormone levels were elevated at 1,008 pg/mL. Uric acid levels and creatine phosphokinase levels were elevated at 16.5 mg/dL and 353 U/L, respectively. Protime was mildly elevated to 15.2 seconds, with an unremarkable international normalized ratio (INR) of 1.2. On gross inspection, the urine sample was pale yellow and cloudy in appearance. The urinalysis showed a specific gravity of 1.012, with pH of 5.0, two plus proteinuria, two plus blood, three plus white blood cell esterase, and positive for leukocytes and red blood cells. Urine culture grew Escherichia coli (100,000 CFU/mL). Serological work-up including human immunodeficiency virus (HIV) antigen/antibodies and hepatitis B core IgM antibody, and urine drug screen were all negative.

**Figure 1:**
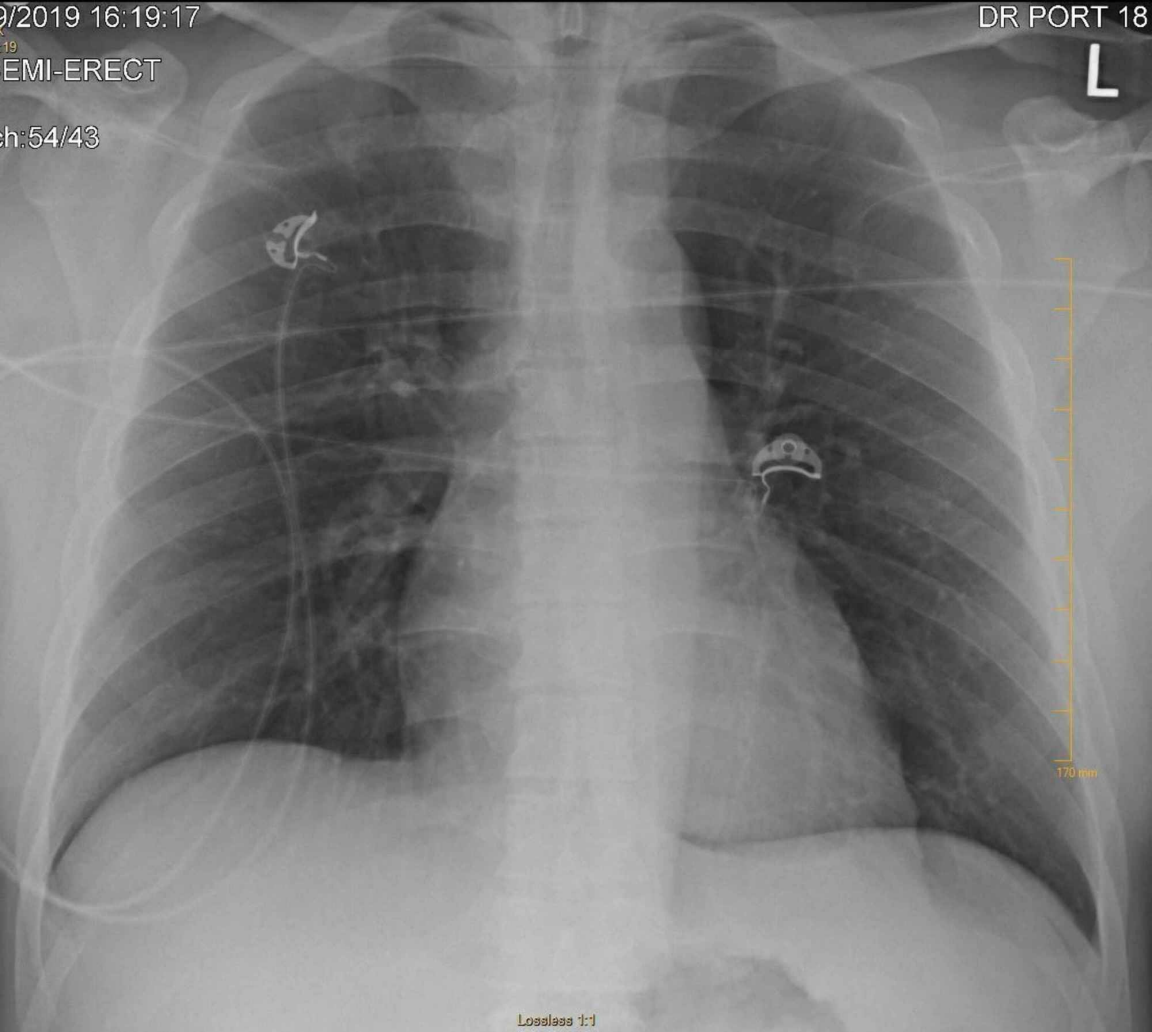
Chest X-ray showing no acute abnormalities.

**Figure 2:**
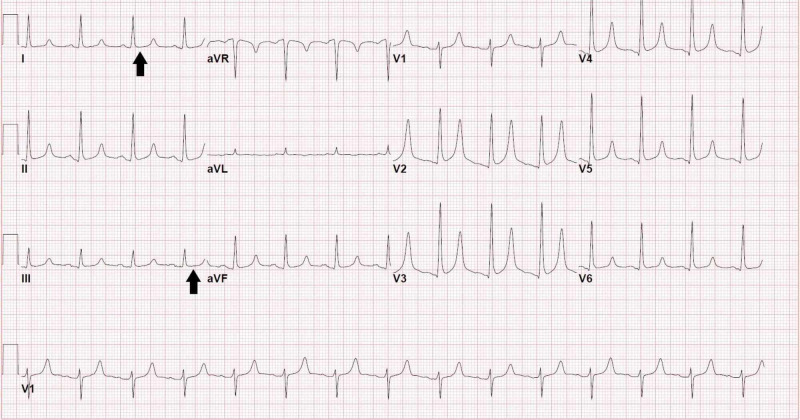
Electrocardiogram showing prolonged QT interval (black arrows).

**Figure 3:**
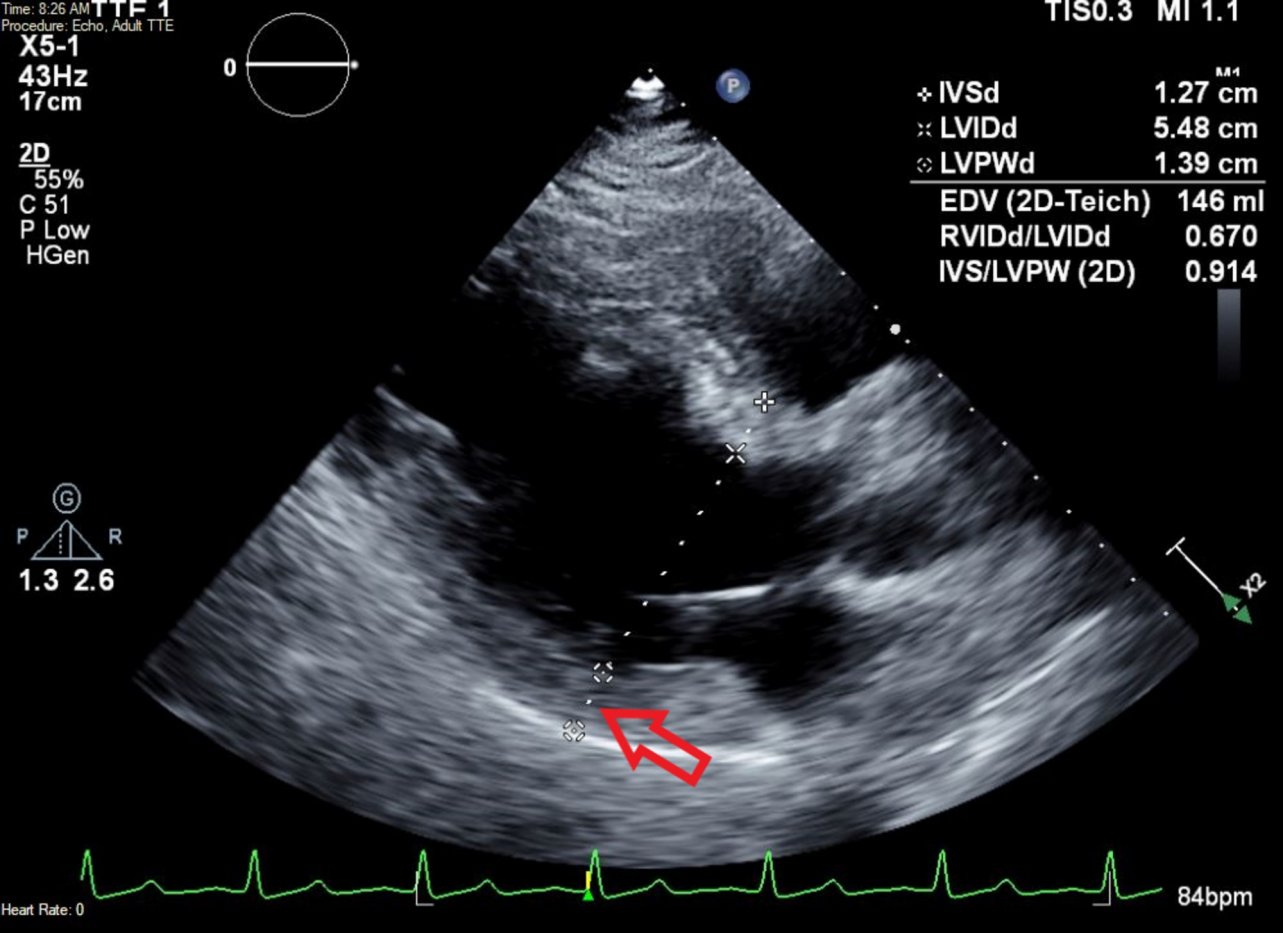
Echocardiogram (parasternal long-axis view) showing thickened end-diastolic left ventricle posterior wall (red arrow).

**Figure 4:**
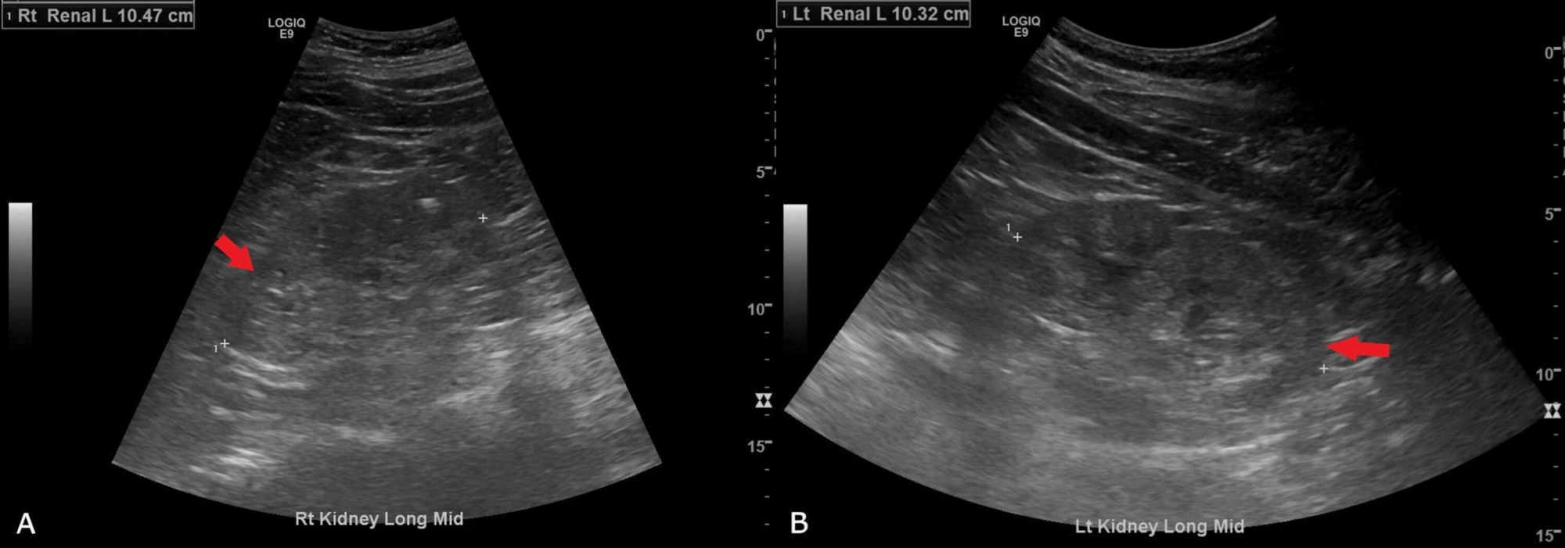
Ultrasound of the right (A) and the left (B) kidney shows increased parenchymal echogenicity and decrease in cortical thickness (red arrows). Measurement of the kidney length is illustrated by ‘+’.

Chest X-ray did not show any signs of fluid overload such as pulmonary edema or pleural effusion (Figure 1). Electrocardiogram showed prolonged QT (QTc 493 ms) (Figure 2) and transthoracic echocardiogram revealed mild left ventricular hypertrophy (Figure 3). Renal ultrasound reported bilaterally increased parenchymal echogenicity and cortical thinning consistent with chronic renal insufficiency (Figure 4).

The patient emergently underwent transfusion with two units of packed red blood cells. HTN was controlled with amlodipine and intravenous hydralazine. His metabolic acidosis was slowly corrected with intravenous bicarbonate infusion. An internal jugular venous hemodialysis catheter was placed, and the patient underwent emergent hemodialysis. With initiation of hemodialysis, bleeding and electrolyte imbalances resolved. During his hospital course, he was also treated with a five-day course of ceftriaxone for urinary tract infection. At discharge, his creatinine level had come down to 15.1 mg/dL, BUN was down to 56 mg/dL, potassium was down to 3.9 mmol/L and hemoglobin was 7.5 g/dL. Eventually, the patient was transitioned to peritoneal dialysis. Unfortunately, due to the patient's non-compliance with dialysis, he has been admitted multiple times over the past year with similar presentations.

## Discussion

Our patient presented with acute renal failure on CKD, anion gap metabolic acidosis, hyperphosphatemia, and hyperparathyroidism likely secondary to renal failure. CKD was thought to be due to chronic uncontrolled HTN causing nephrosclerosis. The patient also had acute blood loss anemia in the setting of anemia of chronic disease. The continuous oozing of blood from the one week old cut on his lips was likely secondary to platelet dysfunction due to uremia. Apart from the presence of minimal symptoms of platelet dysfunction and generalized fatigue, the patient's hemodynamic status seemed fairly stable in the setting of significant uremia and unprecedented creatinine level.

Creatinine is an amino acid compound derived from creatine. It is released into the plasma at a constant rate, freely filtered by the glomerulus, and is not reabsorbed or metabolized by the kidney [4]. Creatinine levels have been traditionally used to assess residual kidney function, but the levels are substantially affected by nutritional status, muscle mass, age, and sex of the patient [5-7]. A retrospective cohort study of 5,000 patients by Fink et al. found an inverse correlation between incident creatinine levels before initiation of dialysis in ESRD patients and mortality [6]. Higher creatinine levels may be associated with lower mortality or increased survivability possibly due to better nutritional status, muscle mass, and functional status of the patient [5,6]. This was the case with our patient who had good nutritional status and muscle mass on presentation.

The exact effect of creatinine levels on human physiology is not completely understood, and there is no established higher end of creatinine level which is compatible with life [5]. But this case along with examples in the literature point toward the fact that it is not the serum creatinine levels that cause fatality but it is the electrolyte imbalance and metabolic derangements associated with CKD/ESRD, which leads to mortality [5].

A literature search of PubMed, Medline, and Google Scholar revealed only four other case reports with a creatinine level higher than that of our patient [2,5,7,8]. Table 1 summarizes the clinical presentation, as well as creatinine and BUN levels of these reported cases. Creatinine measurement employed in our facility’s laboratory fails to record levels greater than 37 mg/dL. This was a limiting factor in our ability to document the exact creatinine level in our patient at presentation, which may possibly be in the range or higher than the other mentioned cases.

**Table 1:** Demography, risk factors, clinical characteristics, and laboratory findings of the cases with extraordinary creatinine levels. BUN, blood urea nitrogen; BP, blood pressure; HR, heart rate (beats per minute); RR, respiratory rate (breaths per minute); SpO_2_, blood oxygen saturation levels; BMI, body mass index

Publication Year	Age/sex	Ethnicity	Presentation	Hemodynamic status	Risk factors	Creatinine (mg/dL)/BUN (mg/dL)	Method	Management	Study
2015	17/male	African-American	Vomiting, headache, respiratory distress, periorbital and lower extremity edema	Afebrile; BP = 179/93 mm Hg; HR = 88; RR = 40; SpO_2_ = 100% on RA	Hypertension, obesity (BMI: 35.49 kg/m^2^)	52/203	Enzymatic method	Hemodialysis	Master Sankar Raj et al. [2]
2014	14/female	African-American	Vomiting, sore throat	Afebrile; BP = 114/56 mm Hg; HR = 88 bpm; RR = 18; SpO_2_ = 98% on RA	Obesity, risperidone and aripiprazole use	43.28/293	Enzymatic method: cobas c 311/501 analyzer	Hemodialysis	Okechuku and Upadhyay [7]
2013	34/male	Unknown	Nausea, vomiting, diarrhea, weight loss, nocturia, ankle swelling, asterixis	BP = 184/93 mm Hg; SpO_2_ = 100% on RA Rest of the vitals were within normal limits	None	53/135	Dilution method	Hemodialysis	Storm et al. [5]
2012	20/male	Unknown	Headache, nausea, hypertension	BP = 190/105 mm Hg; HR = 98 bpm; RR = 24	Hypertension	61.3/245	BioAssay QuantiChrom™ Creatinine Assay Kit [DICT-500]	Hemodialysis	Abuhasna [8]

## Conclusions

Apart from the presence of minimal symptoms of platelet dysfunction and generalized fatigue, the patient's hemodynamic status seemed fairly stable in the setting of significant uremia and unprecedented creatinine. By virtue of this case report, we hope to encourage others to report similar cases and spread awareness regarding the minimal contribution of serum creatinine levels in pathophysiology of renal disease patients.
